# TLR9 and NF-κB Are Partially Involved in Activation of Human Neutrophils by *Helicobacter pylori* and Its Purified DNA

**DOI:** 10.1371/journal.pone.0101342

**Published:** 2014-07-02

**Authors:** Lourdes Alvarez-Arellano, Pedro Cortés-Reynosa, Norma Sánchez-Zauco, Eduardo Salazar, Javier Torres, Carmen Maldonado-Bernal

**Affiliations:** 1 Unidad de Investigación en Enfermedades Infecciosas, Hospital de Pediatría del Instituto Mexicano del Seguro Social, Mexico City, México; 2 Laboratorio de Neuroinmunobiología del Departamento de Medicina Molecular y Bioprocesos, Instituto de Biotecnología de la Universidad Nacional Autónoma de México, Cuernavaca, Morelos, México; 3 Departamento de Biología Celular del Centro de Investigación y Estudios Avanzados, Instituto Politécnico Nacional, Mexico City, México; 4 Laboratorio de Investigación en Inmunología y Proteómica, Hospital Infantil de México Federico Gómez, Mexico City, México; University of Cologne, Germany

## Abstract

*Helicobacter pylori* infection represents one of the most common bacterial infections worldwide. The inflammatory response to this bacterium involves a large influx of neutrophils to the lamina propria of the gastric mucosa. However, little is known about the receptors and molecular mechanisms involved in activation of these neutrophils. In this study, we aimed to determine the role of toll-like receptor 9 (TLR9) in the response of human neutrophils to *H. pylori* and purified *H. pylori* DNA (Hp-DNA). Neutrophils were isolated from the blood of adult volunteers and challenged with either *H. pylori* or Hp-DNA. We found that both, *H. pylori* and Hp-DNA induced increased expression and release of IL-8. Furthermore, we showed that TLR9 is involved in the induction of IL-8 production by *H. pylori* and Hp-DNA. IL-8 production induced by *H. pylori* but not by Hp-DNA was partially mediated by NF-κB. In conclusion, this study showed for first time that both, *H. pylori* and Hp-DNA activate TLR9 and induce a different inflammatory response that leads to activation of neutrophils.

## Introduction

Innate immunity is the first line of defense against microorganisms, which are recognized by members of a specialized family of pattern recognition receptors (PRR) that includes the TLRs. TLRs are type I transmembrane receptors that have an extracellular leucine-rich repeat recognition domain and an intracellular IL-1R-like signaling domain. The activation of TLRs triggers a signaling cascade that leads to the transcription of molecules involved in the inflammatory response, including cytokines, chemokines, adhesion and costimulatory molecules [1]. TLRs recognize a wide range of microorganism-associated molecular patterns including LPS, peptidoglycan, lipoteichoic acid, lipopeptides, flagellin and nucleic acids [2].

*H. pylori* is a Gram-negative bacterium that colonizes the human gastric mucosa and represents one of the most common bacterial infections worldwide. The infection usually leads to chronic asymptomatic inflammation of the gastric wall, although in approximately 10% of individuals it can cause peptic ulcers and in <3% can cause gastric cancer or MALT lymphoma [3].

*H. pylori* infection induces an inflammatory response that includes a large influx of inflammatory cells to the lamina propria of the gastric mucosa [4], and neutrophils are usually the first cells to arrive at the site of infection. In *H. pylori*-associated chronic active gastritis, neutrophils are localized in the lamina propria, within the gastric epithelium and in the foveolar lumen [5]. It has been shown that neutrophils and a strong inflammatory response are essential for the resolution of *H. pylori* infection [6]. In addition, there is a correlation between IL-8 mRNA expression in the gastric wall and the severity of tissue damage [7]. We previously showed that *H. pylori* induced IL-8 production in neutrophils by a mechanism partially dependent on TLR2 and TLR4 [8]. However, it was unknown whether TLR9 in human neutrophils is also responsive to *H. pylori* or purified *H. pylori* DNA (Hp-DNA).

Several studies have demonstrated that *H. pylori* can activate innate immunity via TLRs [9], [10]. TLR2 and TLR4 expressed on BM-derived Mφ were shown to be necessary for *H. pylori*-induced activation of the immune response leading to secretion of IL-6, IL-1β, IL-12 and IL-10, although TLR9 was not required for this process [11]. However, increased expression of TLR9 was demonstrated in gastric tissues of patients infected with *H. pylori*, suggesting an important role of this receptor in the immune response to this infection [5]. In addition, the recognition of microbial DNA by TLR9 is an essential component of the innate immune response to infections [12], and it has been shown that human neutrophils express TLR9 [13], [14]. We aimed to study the role of TLR9 in the response of human neutrophils to *H. pylori* infection and to Hp-DNA. We found that both *H. pylori* and Hp-DNA induced TLR9-dependent IL-8 production and NF-κB activation in human neutrophils.

## Materials and Methods

### Ethics Statement

This research protocol was approved by the Research and Ethics Committee of the Hospital de Pediatría del Centro Médico Nacional sXXI, Instituto Mexicano del Seguro Social (Registration R-2005-3603-50). The peripheral blood of healthy donors was obtained from the Blood Bank at the same Institution. The bags of blood used in the study were destined for waste by the bank of blood, for that reason the letter of consent was not necessary.

### Isolation of human neutrophils

Neutrophils were isolated by density gradient from buffy coats from healthy *H*. *pylori*-seronegative donors, as previously described [8]. They were then resuspended in RPMI 1640 medium (Gibco BRL, Life Technologies, MD) supplemented with 2% fetal bovine serum and penicillin/streptomycin 100 UI/100 µg/mL (Gibco BRL, Life Technologies, MD), and cultured at 37°C in 5% CO_2_. Neutrophil viability was confirmed by Trypan blue dye exclusion before each experiment (Gibco BRL, Life Technologies, MD).

### *H. pylori* culture and isolation of DNA

For stimulation of neutrophils, *H. pylori* strain 26695 was grown on 5% defibrinated sheep blood agar base plates at 37°C in 9% CO_2_. After 24 h, growth was harvested and resuspended in 0.9% saline solution. The OD at 550 nm of the suspension was determined and the concentration adjusted to a final absorbance of 0.750 in 0.9% saline solution.

DNA was isolated using a QIAamp DNA Mini Kit (Qiagen, Valencia, CA) according to the manufacturer's instructions, and quantified with a NanoDrop ND-1000 Spectrophotometer (Wilmington, Delaware). DNA was treated with polymyxin B (5 µg/mg DNA) for 1 h at room temperature. To confirm that activity was due to DNA, isolated DNA was treated with DNase I (Ambion, Austin, TX) for 1 h at 37°C, and degradation was confirmed by electrophoresis in 0.7% agarose gel before testing.

### Expression of IL-8 mRNA

Total RNA was isolated from human neutrophils using TRIzol Reagent (Invitrogen, Carlsbad, CA) according to the manufacturer's instructions, and RT performed using Superscript III RT (Invitrogen, Carlsbad, CA). Expression of IL-8 and GAPDH (endogenous control) mRNAs was tested by real-time PCR using an ABI PRISM 7000 (Applied Biosystems, Foster City, CA) and Taqman reagents following the manufacturer's instructions. The primer sequences used were 5′–AGACAGCAGAGCACACAAGC–3′ and 5′–ATGGTTCCTTCCGGTGTT–3′ for IL-8; 5′–GAAGGTGAAGGTCGGAGTC–3′ and 5′–GAAGATGGTGATGGGATTTC–3′ for GAPDH. IL-8 relative expression was calculated by normalizing to GAPDH using the ΔCT method.

### IL-8 protein quantification

Human neutrophils (1×10^7^ cells) were stimulated with *H. pylori* (1×10^9^ bacteria) or Hp-DNA (1 µg/mL) for 0.5, 1, 3, 6 and 24 h at 37°C in 5% CO_2_. After stimulation, cell-free culture supernatants were harvested and the concentration of IL-8 measured by an ELISA with a detection limit of 4 pg/mL (OptEIA, BD Pharmingen, San Diego, CA) according to the manufacturer's instructions. In each experiment, every sample was tested in duplicate and the results are the average of at least three independent assays.

### Expression of TLR9 in the cell surface

Neutrophils (2.5×10^5^) were stimulated with *H. pylori* (2.5×10^7^ bacteria) or Hp-DNA (1 µg/mL) for 3 h and the cell-surface expression of TLR9 was determined by confocal microscopy and flow cytometry. To confocal microscopy, the neutrophils were fixed with 2% paraformaldehyde in PBS (phosphate buffered saline) and blocking with 10% BSA in PBS for 1 h. Next, the cells were washed and incubated for 1 h with fluorescein isothiocyanate-conjugated anti-TLR9 (Santa Cruz Biotechnology, Santa Cruz, CA) and then stained with TOTO-3 iodide (Invitrogen, Carlsbad, CA) for 10 min in the dark, washed and visualized using a 63X/1.2 W objective and Zeiss LSM510 META confocal microscope (Carl Zeiss). To flow cytometry, treated neutrophils were resuspended in blocking buffer (PBS containing 2% FBS, 2% rabbit serum, 5 mM EDTA and 0.1% sodium azide) and incubated on ice for 1 h. The cells suspension was centrifuged and stained with fluorescein isothiocyanate-conjugated anti-TLR9 Ab (Abcam, Cambridge, UK). The cells were incubated for 15 min in the dark, washed twice with fluorescence-activated cell sorting (FACS) buffer and fixed with 4% paraformaldehyde in PBS for 30 min. Cells were analyzed with a FACScan flow cytometer using a software FACS DIVA (BD, Biosciences).

### Assay for inhibition of TLR9

The involvement of TLR9 in the activation of neutrophils was tested by blocking experiments using a polyclonal Ab that was previously reported with neutralizing activity [15]. Neutrophils (1×10^7^) were first pretreated with human IgG (500 µg/mL) (Aventis Behring GmbH, Marburg, Germany) for 1 h at 37°C in order to saturate FcR and to avoid nonspecific attachment of anti-TLR9. Next, neutrophils were treated with anti-TLR9 polyclonal Ab (clone D-18, 5 µg/mL) (Santa Cruz Biotechnology, Santa Cruz, CA) for 1 h at 37°C, followed by challenge with *H. pylori* (1×10^9^) or Hp-DNA (1 µg/mL) for 24 h, and the production of IL-8 was quantified in culture supernatants by ELISA. We also used an inhibitory ODN (oligonucleotide) (TTAGGG, InvivoGen, San Diego, CA) or chloroquine, an inhibitor of endosomal acidification (Sigma-Aldrich, St. Louis, MO) as inhibitors of TLR9 as follows: neutrophils were preincubated with inhibitory ODN (1 µg/mL) or chloroquine (5 µg/mL) for 1 h and then stimulated with *H. pylori* or Hp-DNA for 24 h. After that, the production of IL-8 was determined in the culture supernatants by ELISA. Each inhibition experiment was run in duplicate in four independent assays and with neutrophils from different blood-donor individuals.

### Activation and inhibition of NF-κB

Neutrophils (1×10^7^) were stimulated with *H. pylori* (1×10^9^) or Hp-DNA (1 µg/mL) for 0.5, 1, 2 or 3 h and nuclear protein extracts were prepared for EMSA as previously described [19]. Briefly, neutrophils were lysed with 10% Igepal (Sigma-Aldrich, St Louis, MO) in Buffer A (10 mM Tris–HCl pH 7.0, 10 mM NaCl, 6 mM MgCl_2_, 10 mM NaF, 1 mM Na_3_VO_4_, 1 mM DTT, 1 mM PMSF), lysates were pelleted at 2,500 rpm for 5 min at 4°C and resuspended in Buffer B (20 mM HEPES pH 7.9, 420 mM NaCl, 1.5 mM MgCl_2_, 0.2 mM EDTA, 1 mM Na_3_VO_4_, 10 mM NaF, 1 mM DTT, 0.2 mM PMSF). Nuclear extracts were recovered by centrifugation at 14,000 rpm for 15 min at 4°C and protein concentration was determined by Bradford protein assay (Bio-Rad, Hercules, CA). For EMSA, double-stranded oligonucleotides containing the consensus sequences for NF-κB DNA-binding site (5′–AGCTAAGGGACTTTCCGCTGGGGACTTTCCAGG–3′ and 5′–AGCTCCTGGAAAGTCCCAGCGGAAAGTCCCTT–3′) were end-labeled with [γ-^32^P] ATP using T4 polynucleotide kinase. The ^32^P-labeled oligonucleotide probe (1 ng) and nuclear extracts (7 µg) were incubated in a binding buffer (3 µg of poly (dI-dC), 0.25 M HEPES pH 7.5, 0.6 M KCl, 50 mM MgCl_2_, 1 mM EDTA, 7.5 mM DTT and 9% glycerol) for 20 min at 4°C. The specificity of the binding reaction was determined by preincubation of nuclear extracts either with 100-fold excess of unlabeled NF-κB probe or with an irrelevant oligonucleotide (5′–ACGTGTGATGAAATGCTAGGCGATC–3′ and 5′–GATCGCCTAGCATTTCATCACACGT–3′). The samples were separated on a 4% polyacrylamide gel in 0.5× Tris-borate-EDTA buffer. After electrophoresis, the gels were dried and exposed to radiography film. The OD of bands was obtained using Labworks Analysis Software (UVP Inc., Upland, CA).

NF-κB inhibition assays were performed using PDTC (pyrrolidine dithiocarbamate) (Sigma-Aldrich, St. Louis, MO) and DHMEQ (dehydroxymethyl epoxyquinomycin). Neutrophils (5×10^6^) were preincubated for 1 h with PDTC (300 µM) or DHMEQ (5 µg/mL) and then stimulated for 24 h with *H. pylori* (5×10^8^) or Hp-DNA (1 µg/mL). Finally, the production of IL-8 in culture supernatant was quantified by ELISA.

### Statistical analysis

Means were compared by Students *t*-test, the Mann–Whitney or Dunnett's test. Differences were considered statistically significant at *p*<0.05. The Sigma Stat software (Jandel Scientific Software, CA) was used for analysis.

## Results

### Isolated Hp-DNA induces IL-8 production in human neutrophils

**Figure 1:**
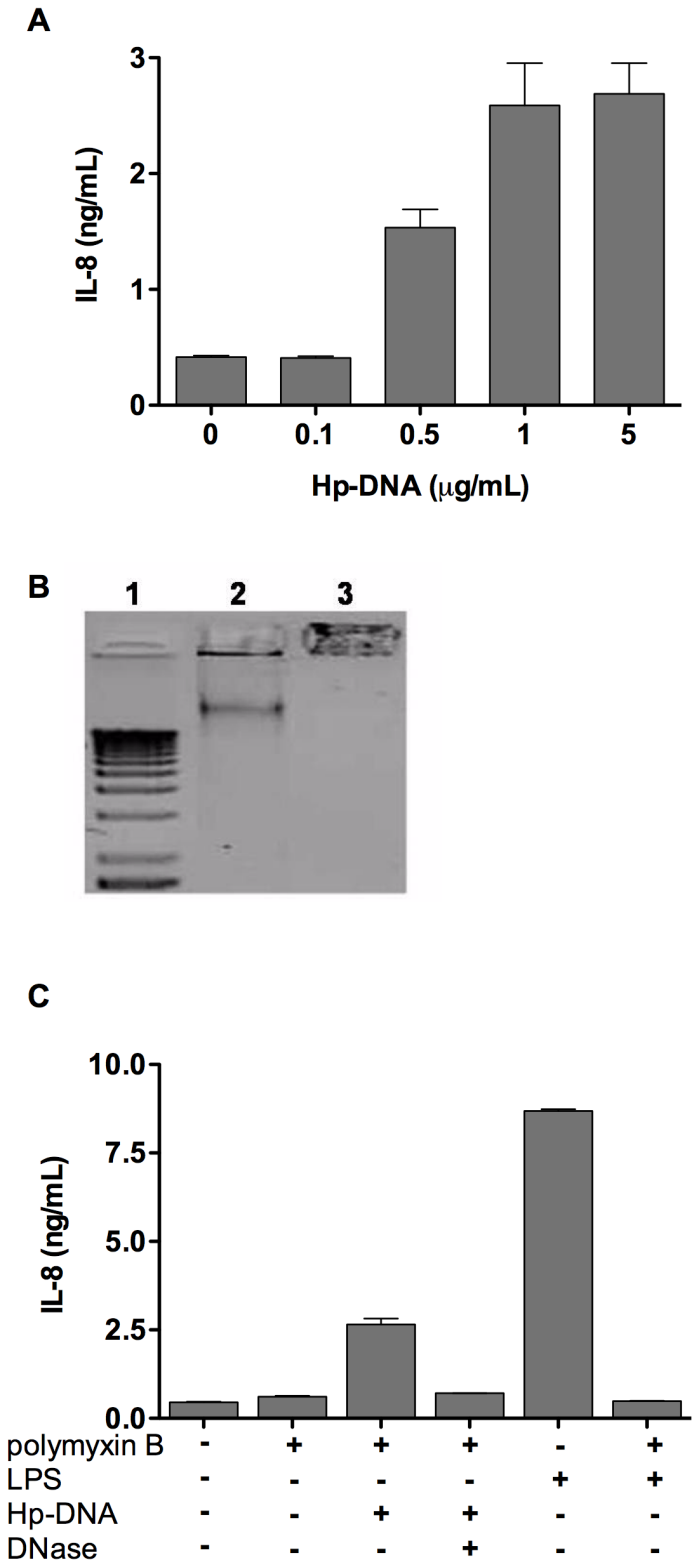
Hp-DNA induces IL-8 production in human neutrophils. Neutrophils were stimulated with Hp-DNA (0.1, 0.5, 1.0 and 5.0 µg/mL) after it had been preincubated for 1 h with polymyxin B (5 µg/1 µg of DNA) to eliminate LPS contamination. (**A**) After 24 h, IL-8 released to supernatants was measured by ELISA. (B) Electrophoresis in 0.7% agarose gel of Hp-DNA (lane 2), Hp-DNA obtained after digestion with DNase I (lane 3) and a molecular weight marker (lane 1). (C) Neutrophils were cultured in the presence or absence of Hp-DNA (1 µg/mL), *E. coli*-LPS (1 µg/mL), polymyxin B and DNase. After 24 h IL-8 released to supernatants was measured by ELISA. Data represent the mean + SD of three independent experiments.

Unmethylated CpG, which is found in large quantities in the DNA of bacteria and viruses, is an agonist of TLR9, so we investigated whether Hp-DNA is capable of activating human neutrophils via TLR9. First, Hp-DNA was preincubated with polymyxin B to inhibit any contaminating LPS and used at different concentrations to challenge human neutrophils, followed by evaluation of IL-8 production. We observed a dose-dependent response with maximum IL-8 production obtained after challenge with 1 µg/mL of Hp-DNA (Fig. 1A); this concentration was used in all subsequent experiments. In contrast, IL-8 production induced by Hp-DNA digested with DNase was similar to that observed in mock-stimulated control cells (Figs 1B and 1C). These results suggest that Hp-DNA induces IL-8 production in human neutrophils.

### Time-dependent secretion and expression of IL-8 induced by *H. pylori* and Hp-DNA

**Figure 2:**
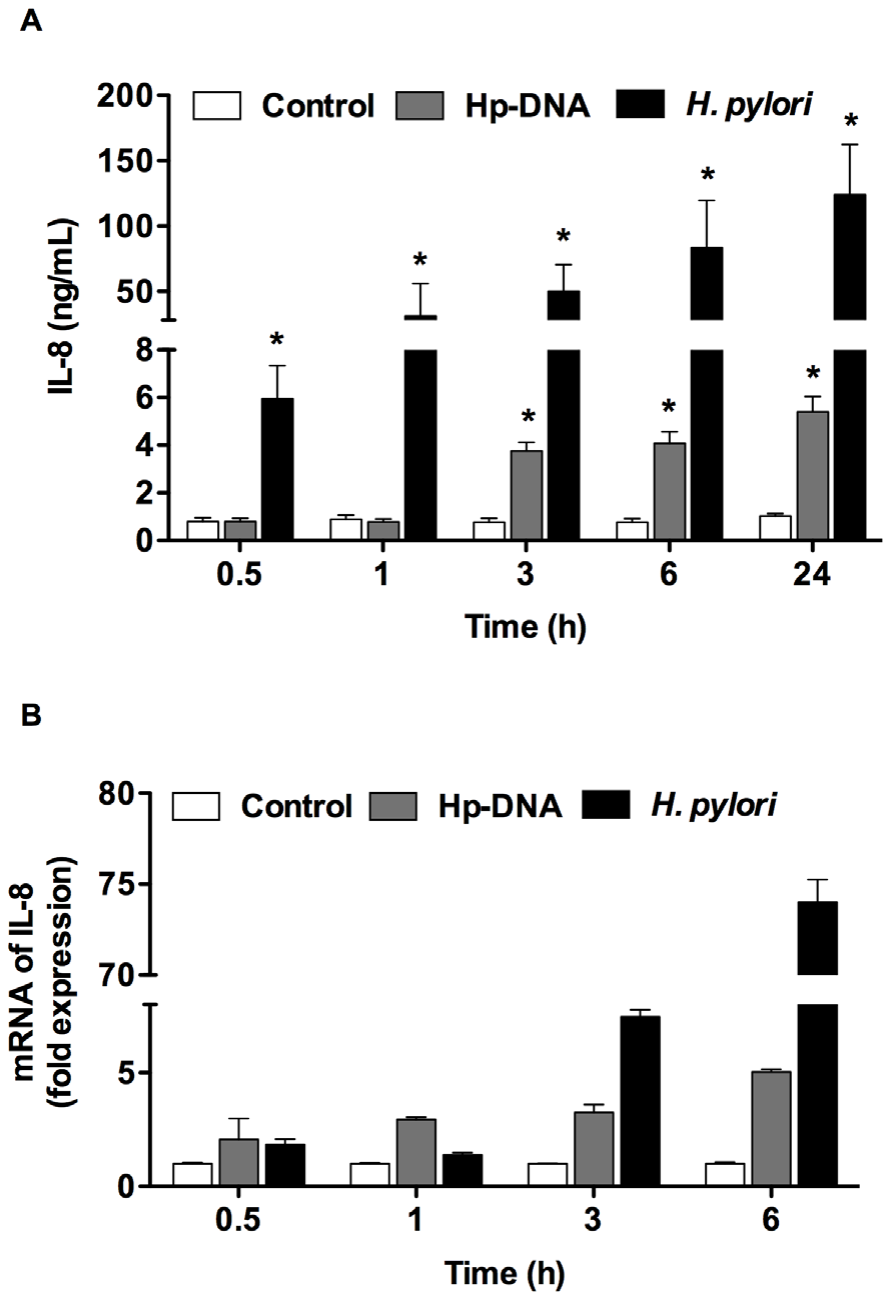
*H. pylori* and Hp-DNA increase both IL-8 protein and IL-8 mRNA. (A) Neutrophils were incubated in the presence or absence of *H. pylori* or Hp-DNA for 0.5, 1, 3, 6 and 24 h and the IL-8 released to supernatants measured by ELISA. (B) For mRNA IL-8 expression, the cells were lysed and IL-8 mRNA expression was determined by real-time PCR. Data represent the mean + SEM of three experiments (A) and a representative result of three independent experiments (B). *, *p*<0.01.

IL-8 plays an important role in the inflammatory process in *H. pylori* infection, recruiting neutrophils and monocytes to the lamina propria of the gastric epithelium. We examined the kinetics of IL-8 protein secretion and mRNA expression in neutrophils that were challenged with either Hp-DNA or *H. pylori*. As shown in Figure 2A, released of IL-8 started earlier with *H. pylori* challenge (after 0.5 h) than with Hp-DNA (after 3 h). The level of IL-8 protein production was also higher in neutrophils stimulated with *H. pylori* (5.9–123.9 ng/mL) than in those stimulated with Hp-DNA (0.78–5.4 ng/mL) (Fig. 2A). DNA and LPS of *E. coli* (used as positive controls) also induced production of IL-8 (Fig. S1), although induction was considerably greater (50 ng/mL) than that induced by the Hp-DNA, but lower than that induced by *H. pylori*. IL-8 mRNA expression also increased in a time-dependent manner with a significant expression after 3 h with either Hp-DNA or *H. pylori* (Fig. 2B). These results indicate that with *H. pylori* there is an increased secretion of IL-8 at early times probably by the release of preformed IL-8 [16], since no increase of mRNA-IL-8 is observed at this time points (0.5 and 1.0 h). Whereas with both, Hp-DNA and *H. pylori* there is an increase in IL-8 after 3 h, which might result from de novo synthesis.

### TLR9 is involved in the *H. pylori*- and Hp-DNA-induced production of IL-8 by human neutrophils

**Figure 3:**
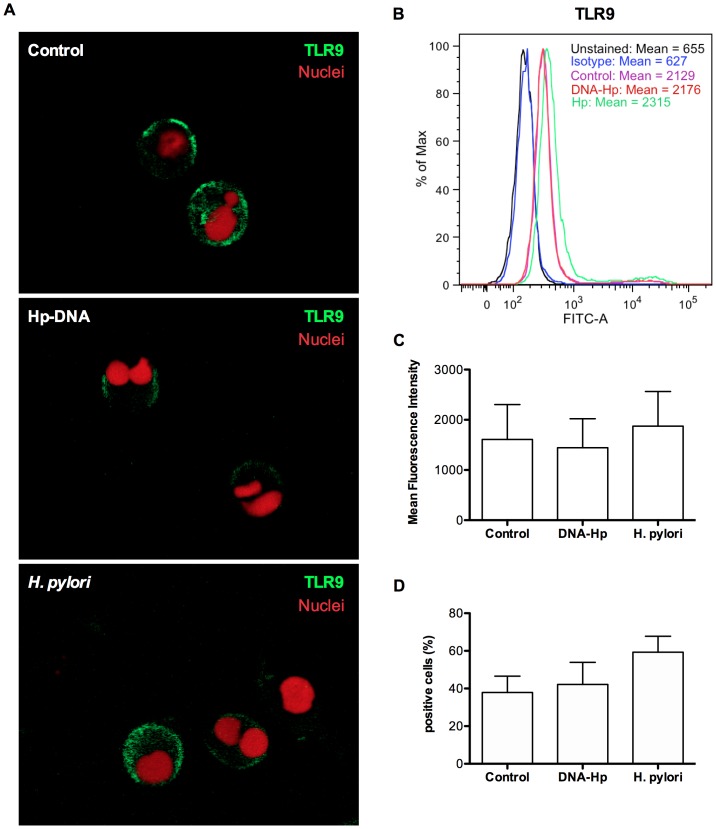
Expression of TLR9 on the surface of human neutrophils stimulated with Hp-DNA and *H. pylori* . Neutrophils (2.5×10^5^) were incubated in the presence of Hp-DNA (1 µg/mL) or *H. pylori* (2.5×10^7^ bacteria) or unstimulated (control) for 3 h and the TLR9 expression were analyzed by confocal microscopy (**A**) and by flow cytometry (**B–D**). For confocal microscopy, neutrophils were stained with fluorescein isothiocyanate-conjugated anti-TLR9 Ab (green) and nuclei stained with TOTO-3 iodide (red); and for flow cytometry they were staining with FITC-conjugated anti-TLR9 Ab (purple, red and green histograms); and isotype control (blue histogram) and unstained neutrophils were included (black line), the MFI data is shown in **B**. This figure represents one of three (**A**) and six (**B**) independent experiments performed. The MFI and positive cells in % data are represented as mean + SD of six independent experiments (**C** and **D** respectively).

**Figure 4:**
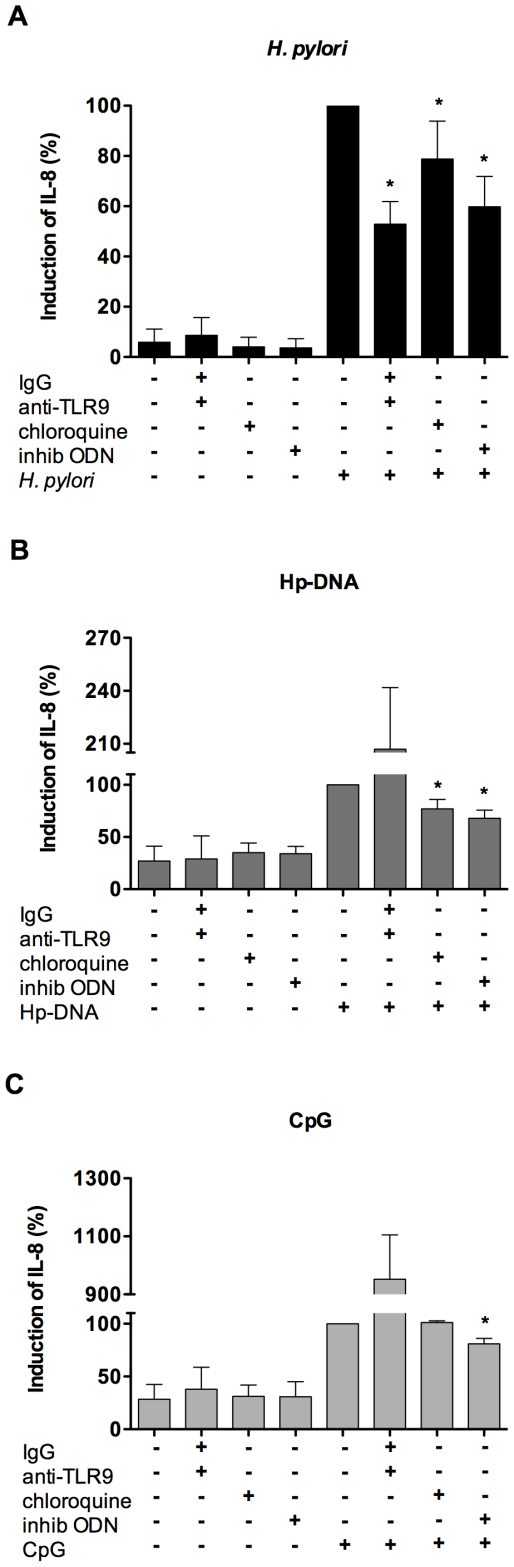
TLR9 is involved in the production of IL-8 induced by *H. pylori* and Hp-DNA. Neutrophils (1×10^7^) were first pretreated in the presence or absence of human IgG (500 µg/mL), and then treated with anti-TLR9 Ab (5 µg/mL), chloroquine (5 µg/mL) or inhibitor ODN (1 µg/mL) for 1 h, and finally challenged with *H. pylori* (1×10^8^) (A), Hp-DNA (1 µg/mL) (B) or CpG (1 µg/mL) (C) for 24 h and then the IL-8 production in the culture supernatant was determined by ELISA. The production of IL-8 induced by *H. pylori*, Hp-DNA and CpG was considered as 100% and the corresponding induction with pretreatments was calculated referring to this value. Data represent the mean + SD of at least three independent experiments. *, *p*<0.05.

We showed previously that *H. pylori* increased TLR2 and TLR4 expression and that *H. pylori*-induced production of IL-8 in neutrophils was partially TLR2 and TLR4-dependent suggesting the involvement of other receptors [8]. In this work, we determined the expression of TLR9 in neutrophils challenged with either Hp-DNA or *H. pylori*. Our results show that TLR9 is constitutively expressed on the surface of unstimulated neutrophils (control) and that its expression did not change in neutrophils challenged with either Hp-DNA or *H. pylori* (Fig. 3A–D). To study the role of TLR9 in IL-8 production induced by *H. pylori*, human neutrophils were pre-incubated with neutralizing anti-TLR9 Ab, chloroquine or inhibitory oligonucleotide (inhibitory ODN, and then stimulated with *H. pylori* or Hp-DNA. We found that *H. pylori*-induced IL-8 production decreased by 47%, 21% and 40%, respectively, suggesting that TLR9 is involved in the induction of IL-8 production (Fig. 4A and S2B). In Fig. 4A we show the results of IgG and anti-TLR9 together and in Fig. S2B we show them separately. When neutrophils were stimulated with Hp-DNA, the pre-incubation with anti-TLR9 did not inhibit but rather increased IL-8 production (Fig. 4B). This result is difficult to explain, probably other receptors such as RAGE (receptor for advanced glycation end-products) [17], which are expressed on neutrophils [18], may also recognize the Hp-DNA and increase IL-8 production. In contrast, pre-incubation with chloroquine or inhibitory ODN, reduced IL-8 production (*p*<0.05) by about 23% and 28%, respectively, suggesting a partial involvement of TLR9 (Fig. 4B). CpG was used as a positive control and as expected, pre-incubation with anti-TLR9 or chloroquine (5 µg/mL) did not inhibit IL-8 production, whereas pre-incubation with highest concentration of chloroquine (10 µg/mL) decreased IL-8 production (Fig. 4C and Fig. S2A), pre-incubation with inhibitory ODN reduced IL-8 production by 19% (*p*<0.05) (Fig. 4C). In addition, as expected IL-8 production was not affected by chloroquine pre-incubation in neutrophils stimulated with *E. coli*-LPS (1.0 µg/mL) for 24 h (data not shown). Overall, these findings suggest that *H. pylori* and Hp-DNA induced IL-8 production via TLR9, but also indicate the existence of other receptors involved in neutrophil activation induced by *H. pylori* and Hp-DNA.

### NF-κB is involved in the activation of human neutrophils by *H. pylori* and Hp-DNA

**Figure 5:**
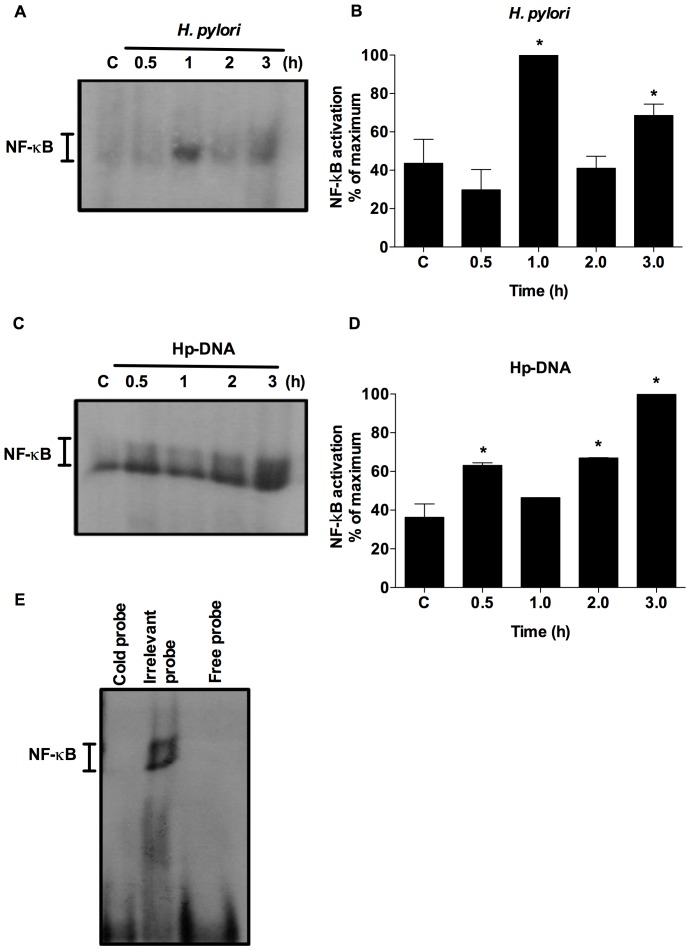
NF-κB activation induced in human neutrophils by *H. pylori* and Hp-DNA. Neutrophils were stimulated with *H. pylori* (**A**) or Hp-DNA (**C**) for the indicated times and nuclear extracts were prepared and NF-κB activation was analyzed by EMSA. Autoradiograms show representative results of three independent experiments. Corresponding densitometric analysis was also performed (**B**, **D**). Results are expressed as percentage of the maximal NF-κB activation observed considered as 100%. The bars represent the mean + SEM of three or four independent experiments. *, *p*<0.05. Controls were performed for nonspecific binding of NF-κB probe (**E**).

NF-κB is an important transcription factor involved in various cellular processes including the induction of transcription of inflammation-related genes. We used EMSA to assess whether *H. pylori* and Hp-DNA activate NF-κB in human neutrophils. Our results showed that *H. pylori* activated NF-κB 1 h after infection (Figs. 5A–B). A similar kinetic was observed with Hp-DNA, although with different timing, here NF-κB activation increased after 0.5 h, then decreased after 1.0 h and increased again after 2 h of stimulation (Figs. 5C and 5D). Although this activation decreased in initial time, probably due to a negative feedback or gene expression control mechanisms, as previously reported [19], [20]. To confirm the specificity of NF-κB binding, we used an unlabeled (cold) probe and an irrelevant probe and found that, as expected, the cold probe but not the irrelevant probe inhibited NF-κB binding (Fig. 5E).

**Figure 6:**
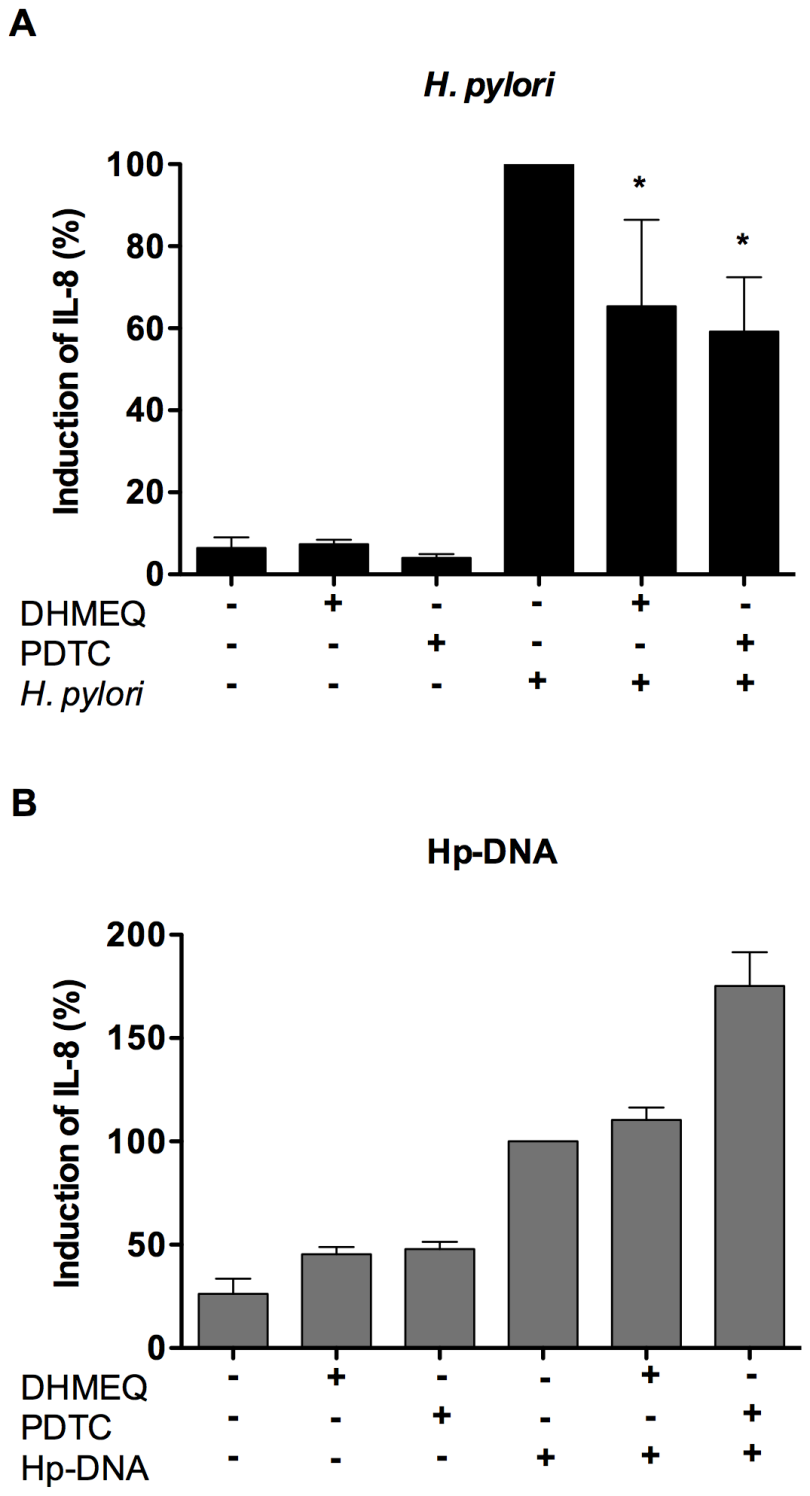
Effect of NF-κB inhibitors on IL-8 production in human neutrophils. Neutrophils (5×10^6^) were preincubated in the presence or absence of NF-κB inhibitors DHMEQ (5 µg/mL) and PDTC (300 µM) for 1 h, then stimulated with *H. pylori* (**A**) or Hp-DNA (**B**) for 24 h and IL-8 production was determined in the culture supernatant by ELISA. Results are expressed as percentage of induction of IL-8, with the concentration of IL-8 induced by *H. pylori* (**A**) or Hp-DNA (**B**) considered as 100% and the rest of the concentrations calculated relative to this value. Data represent the mean + SD of three independent experiments. *, *p*<0.05.

We then evaluated whether IL-8 production induced by *H. pylori* and Hp-DNA was dependent on NF-κB activation. Neutrophils were preincubated with two inhibitors of NF-κB activation, DHMEQ and PDTC, followed by stimulation with *H. pylori* or Hp-DNA, and then IL-8 protein production was determined in the supernatant. Our results showed that preincubation with either DHMEQ or PDTC inhibited *H. pylori*-induced IL-8 production by about 35% and 41%, respectively (Fig. 6A), which suggests that IL-8 induction is partially dependent on NF-κB activation, although other pathways may also participate. In contrast, IL-8 induced by Hp-DNA was not inhibited by DHMEQ or PDTC; rather, these compounds increased IL-8 production (Fig. 6B). These results indicate that NF-κB participates in the induction of IL-8 production by *H. pylori* but not by Hp-DNA.

## Discussion

Neutrophils are thought to be the first cells of the innate immune system to arrive at the lamina propria of the gastric mucosa after infection with *H. pylori*, and have been shown to be essential in the resolution of the infection [6]. Neutrophils express most TLRs, which play important roles in the immune response to pathogens. The results of this study demonstrate that human neutrophils constitutively express functional TLR9 on their surface, which is in agreement with our previous report [21] that was confirmed later by other group [22].

During *H. pylori* infection, IL-8 plays a major part because of its role as a chemoattractant and through its participation in the development of gastric cancer [23], [24]. Previous studies have shown that DNA from *E. coli* activates human neutrophils, inducing the production of IL-8, IL-6, reactive nitrogen species and adhesion molecules [25]–[28]. Our results showed that *H. pylori* and Hp-DNA also induce IL-8 production by human neutrophils, although the induction was higher with *H. pylori* than with Hp-DNA, probably due to the synergistic cooperation between the different PRRs that might to recognize different pathogen-associated molecular patterns in *H. pylori* [29].

Studies have shown that bacterial DNA does not need to be internalized to activate human neutrophils and HEK293 cells [*28,30*], and that activation of neutrophils by bacterial DNA is mediated by a mechanism that does not require the participation of TLR9 [*31*]. In contrast, in this study we present evidence that suggests that Hp-DNA induces IL-8 production by human neutrophils. In addition, the neutralization assays suggest that a fraction of the IL-8 induced by Hp-DNA might be via TLR-9, since the low inhibition by both, cloroquine and ODN (around 25%) was highly reproducible in three independent assays. Our results are consistent with a recent study *showing that in mouse BM-derived DC, Hp-DNA is recognized by TLR9, leading to induction of IL-6 and IL-12 secretion* [*32*] *.* DNA may become available to neutrophils after bacterial lysis or by the natural mechanisms of DNA release for transformation processes in bacterial cultures or in biofilms [33]–[36]. Although it is hard to determine the amount of DNA released *in vivo*, we argue that the TLRs expressed on the surface of phagocytic cells contribute to the process of phagocytosis, which could increase the amount of available DNA inside the cells. To our knowledge, there is no data on DNA liberated during a natural infection, although we would expect this to happen with the *H. pylori* population dying in the gastric mucosa. In fact, it is assumed that *in vivo* transformation is one of the main events explaining the high gene's sequence diversity observed in *H. pylori* [36].

Several studies have demonstrated the importance of NF-κB in the inflammatory process during *H. pylori* infection [37], [38]. *H. pylori* injects peptidoglycan into epithelial cells that is recognized by NOD1, inducing NF-κB activation and IL-8 production [39]. We found that *H. pylori* triggers the activation of NF-κB and that this activation leads to the production of IL-8. TLR9 stimulation in neutrophils leads to activation of the MAP-kinase, PI3-kinase and N-terminal Jun-kinase pathways, and activation of NF-κB and AP-1 [40]. In contrast, although Hp-DNA also activated NF-κB, this activation was not associated with induction of IL-8 production. These results suggest that Hp-DNA can induces IL-8 production using *other transcription factors such as AP*-1 or *NF*-IL-6 [41].

In conclusion, we show that in human neutrophils, *H. pylori* induce activation of TLR9, which leads to an increase in IL-8 production, via NF-κB activation. *In contrast, Hp-DNA also induces* activation of TLR9, but leads to a lower increase in IL-8 production. In addition, the activation of NF-κB by Hp-DNA is not involved in IL-8 production. *In conjunction, these results suggest a role for* neutrophil stimulation via *TLR9 and NF-κB in the initiation of the inflammatory response during* *H. pylori* *infection.*

## Supporting Information

Figure S1 ***H. pylori*** **and Hp-DNA can induce IL-8 production in human neutrophils.** Neutrophils (5×10^6^) were incubated in the presence or absence of *H. pylori* (5×10^8^), Hp-DNA (1 µg/mL), *E. coli*-DNA (100 ng/mL) or *E. coli*-LPS (1 µg/mL) for 24 h and the IL-8 released to supernatants was measured by ELISA. Hp-DNA and *E. coli*-DNA were isolated using a QIAamp DNA Mini Kit (Qiagen, Valencia, CA) according to the manufacturer's instructions, and quantified with a NanoDrop ND-1000 Spectrophotometer (Wilmington, Delaware). Both DNAs were treated with polymyxin B (5 µg/mg DNA) for 1 h at room temperature. Data represent the mean + SD of three independent experiments.
(TIF)

Figure S2 TLR9 is partially involved in the production of IL-8 induced by ***H. pylori*** **,** **Hp-DNA and CpG.** (**A**) Neutrophils (5×10^6^) were pretreated with 1, 5 and 10 µg/mL of chloroquine for 1 h followed by stimulation with Hp-DNA (1 µg/mL) or CpG (1 µg/mL) for 24 h and then the IL-8 production in the culture supernatant was determined by ELISA. The production of IL-8 is shown in ng/mL. Data represent the mean + SD of three independent experiments. Dunnett's test, *, *p*<0.05. (**B**) Neutrophils (5×10^6^) were pretreated in the presence or absence of human IgG (500 µg/mL) or anti-TLR9 Ab (5 µg/mL) for 1 h followed by stimulation with *H. pylori* (5×10^8^) for 24 h and then the IL-8 production in the culture supernatant was determined by ELISA.
(TIF)
