# Supplementary figures and images for: TLR9 and NF-κB Are Partially Involved in Activation of Human Neutrophils by Helicobacter pylori and Its Purified DNA
Source: PLoS One. 2014 Jul 2;9(7):e101342. doi: 10.1371/journal.pone.0101342 (PMC4079333; doi:10.1371/journal.pone.0101342)

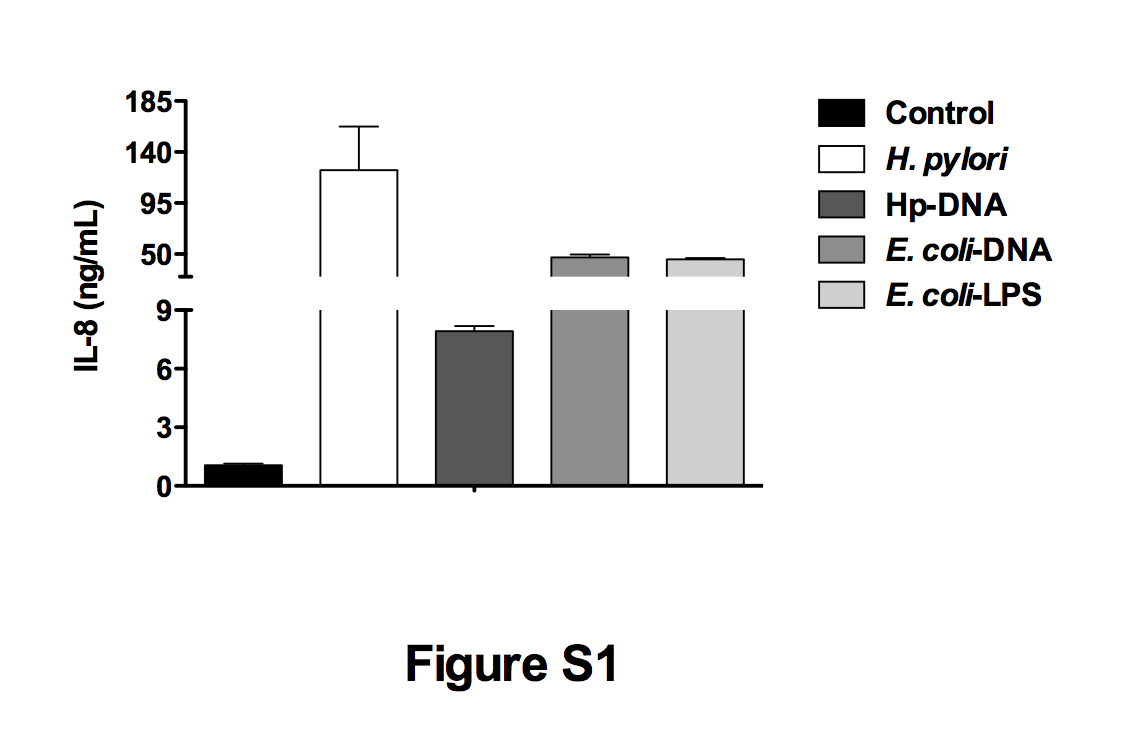

Supplement: Figure S1 — H. pylori and Hp-DNA can induce IL-8 production in human neutrophils. Neutrophils (5×10^6^) were incubated in the presence or absence of H. pylori (5×10^8^), Hp-DNA (1 µg/mL), E. coli-DNA (100 ng/mL) or E. coli-LPS (1 µg/mL) for 24 h and the IL-8 released to supernatants was measured by ELISA. Hp-DNA and E. coli-DNA were isolated using a QIAamp DNA Mini Kit (Qiagen, Valencia, CA) according to the manufacturer's instructions, and quantified with a NanoDrop ND-1000 Spectrophotometer (Wilmington, Delaware). Both DNAs were treated with polymyxin B (5 µg/mg DNA) for 1 h at room temperature. Data represent the mean + SD of three independent experiments. (TIF) [file pone.0101342.s001.tif]

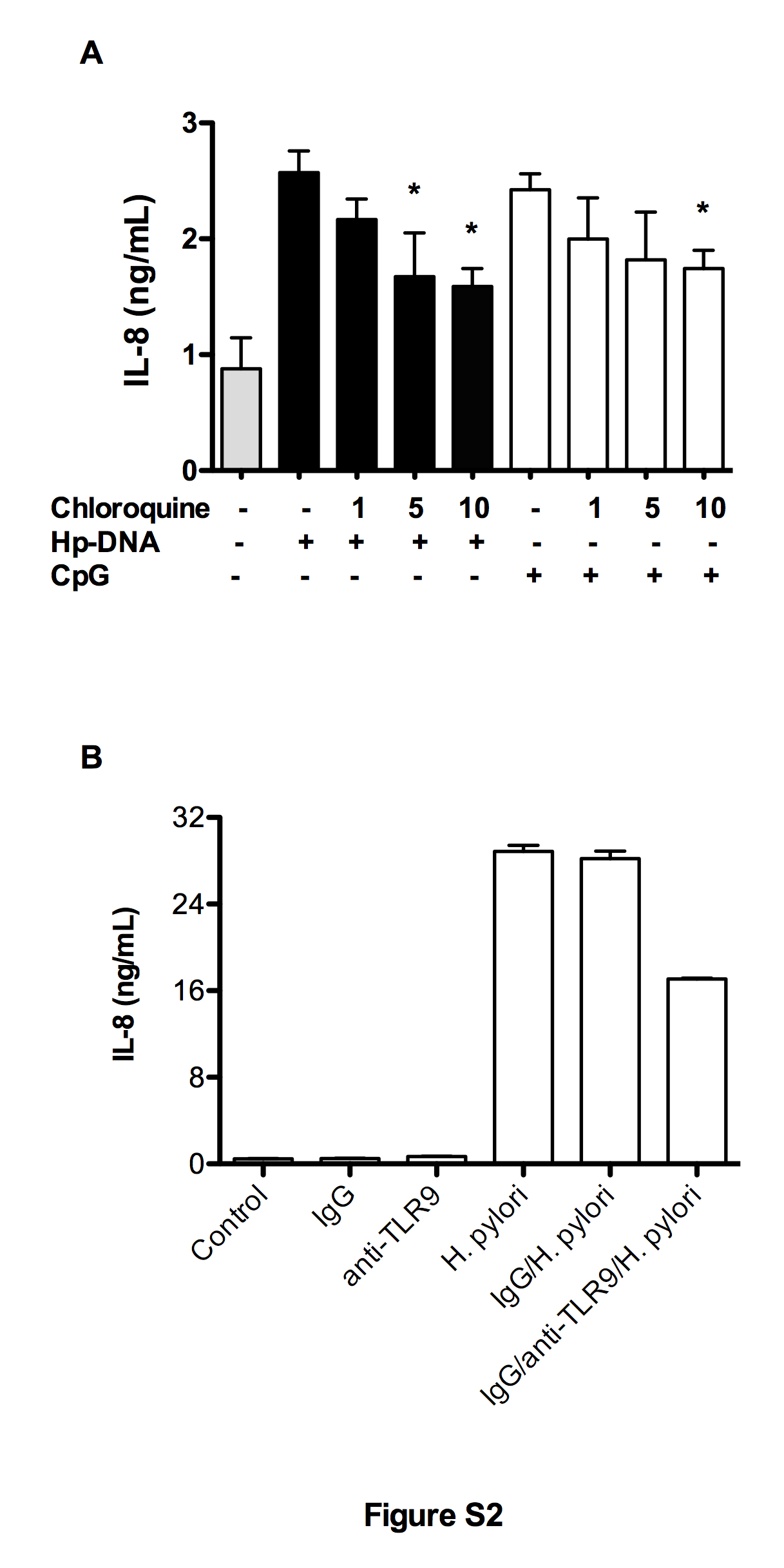

Supplement: Figure S2 — TLR9 is partially involved in the production of IL-8 induced by H. pylori , Hp-DNA and CpG. (A) Neutrophils (5×10^6^) were pretreated with 1, 5 and 10 µg/mL of chloroquine for 1 h followed by stimulation with Hp-DNA (1 µg/mL) or CpG (1 µg/mL) for 24 h and then the IL-8 production in the culture supernatant was determined by ELISA. The production of IL-8 is shown in ng/mL. Data represent the mean + SD of three independent experiments. Dunnett's test, *, p<0.05. (B) Neutrophils (5×10^6^) were pretreated in the presence or absence of human IgG (500 µg/mL) or anti-TLR9 Ab (5 µg/mL) for 1 h followed by stimulation with H. pylori (5×10^8^) for 24 h and then the IL-8 production in the culture supernatant was determined by ELISA. (TIF) [file pone.0101342.s002.tif]
